# Spatiotemporal Processing in Crossmodal Interactions for Perception of the External World: A Review

**DOI:** 10.3389/fnint.2015.00062

**Published:** 2015-12-22

**Authors:** Souta Hidaka, Wataru Teramoto, Yoichi Sugita

**Affiliations:** ^1^Department of Psychology, Rikkyo UniversitySaitama, Japan; ^2^Department of Psychology, Kumamoto UniversityKumamoto, Japan; ^3^Department of Psychology, Waseda UniversityTokyo, Japan

**Keywords:** crossmodal interaction, spatial processing, temporal processing, spatiotemporal processing, motion processing, neural representations

## Abstract

Research regarding crossmodal interactions has garnered much interest in the last few decades. A variety of studies have demonstrated that multisensory information (vision, audition, tactile sensation, and so on) can perceptually interact with each other in the spatial and temporal domains. Findings regarding crossmodal interactions in the spatiotemporal domain (i.e., motion processing) have also been reported, with updates in the last few years. In this review, we summarize past and recent findings on spatiotemporal processing in crossmodal interactions regarding perception of the external world. A traditional view regarding crossmodal interactions holds that vision is superior to audition in spatial processing, but audition is dominant over vision in temporal processing. Similarly, vision is considered to have dominant effects over the other sensory modalities (i.e., visual capture) in spatiotemporal processing. However, recent findings demonstrate that sound could have a driving effect on visual motion perception. Moreover, studies regarding perceptual associative learning reported that, after association is established between a sound sequence without spatial information and visual motion information, the sound sequence could trigger visual motion perception. Other sensory information, such as motor action or smell, has also exhibited similar driving effects on visual motion perception. Additionally, recent brain imaging studies demonstrate that similar activation patterns could be observed in several brain areas, including the motion processing areas, between spatiotemporal information from different sensory modalities. Based on these findings, we suggest that multimodal information could mutually interact in spatiotemporal processing in the percept of the external world and that common perceptual and neural underlying mechanisms would exist for spatiotemporal processing.

## Introduction

In our daily life, we receive dynamic inputs to multiple modalities from, for example, moving cars, the face of a friend with whom we are conversing, and so on. While a large amount of inputs continue to change uniquely in each sensory modality, we can perceive them as integrated, coherent objects, or scenes. Our perceptual systems appropriately and flexibly associate and integrate these inputs (Ernst and Bülthoff, 2004), thus enabling us to establish coherent and robust percepts of the external world in our brains.

Research regarding crossmodal perception/interactions and their underlying mechanisms has garnered much interest in the last few decades. The number of studies related to these issues has risen dramatically (Murray et al., 2013; Van der Stoep et al., 2015). Many researchers have investigated how multiple sensory inputs are integrated/associated in our perceptual systems. They have focused on spatial and temporal integration/association rules (Calvert et al., 2004; Stein, 2012), as well as attentional (see Driver and Spence, 1998 for a review) and neural mechanisms (see Stein and Meredith, 1993; Driver and Noesselt, 2008; Stein and Stanford, 2008 for review). In addition to these studies, crossmodal interactions in the *spatiotemporal* domain (i.e., motion processing) have also been investigated (see Soto-Faraco et al., 2003, 2004a, for review). A traditional view holds that vision is superior to audition in spatial processing, while audition is dominant over vision in temporal processing (Welch and Warren, 1986). Similarly, in spatiotemporal processing, visual information is considered predominant over information from other sensory modalities (Soto-Faraco et al., 2004a). However, recent studies have demonstrated that sound can have a driving effect on visual motion perception (e.g., Hidaka et al., 2009). Moreover, studies regarding audio-visual perceptual associative learning have reported that, after an association is established between sounds and visual motion, sounds without spatial information can trigger visual motion perception (e.g., Teramoto et al., 2010a). Other sensory modalities such as motor action or smell have also exhibited similar driving effects on visual motion perception (e.g., Keetels and Stekelenburg, 2014).

In this way, the findings regarding spatiotemporal processing of crossmodal interactions have been updated in recent years. Here, we summarize past and recent findings of spatiotemporal processing in crossmodal interactions. First, we briefly review some key findings of spatial and temporal processing in crossmodal interactions. Then, we focus on the literature on spatiotemporal processing in crossmodal interactions, including psychophysical and brain imaging findings.

## Crossmodal interactions in spatial domain

**Figure 1:**
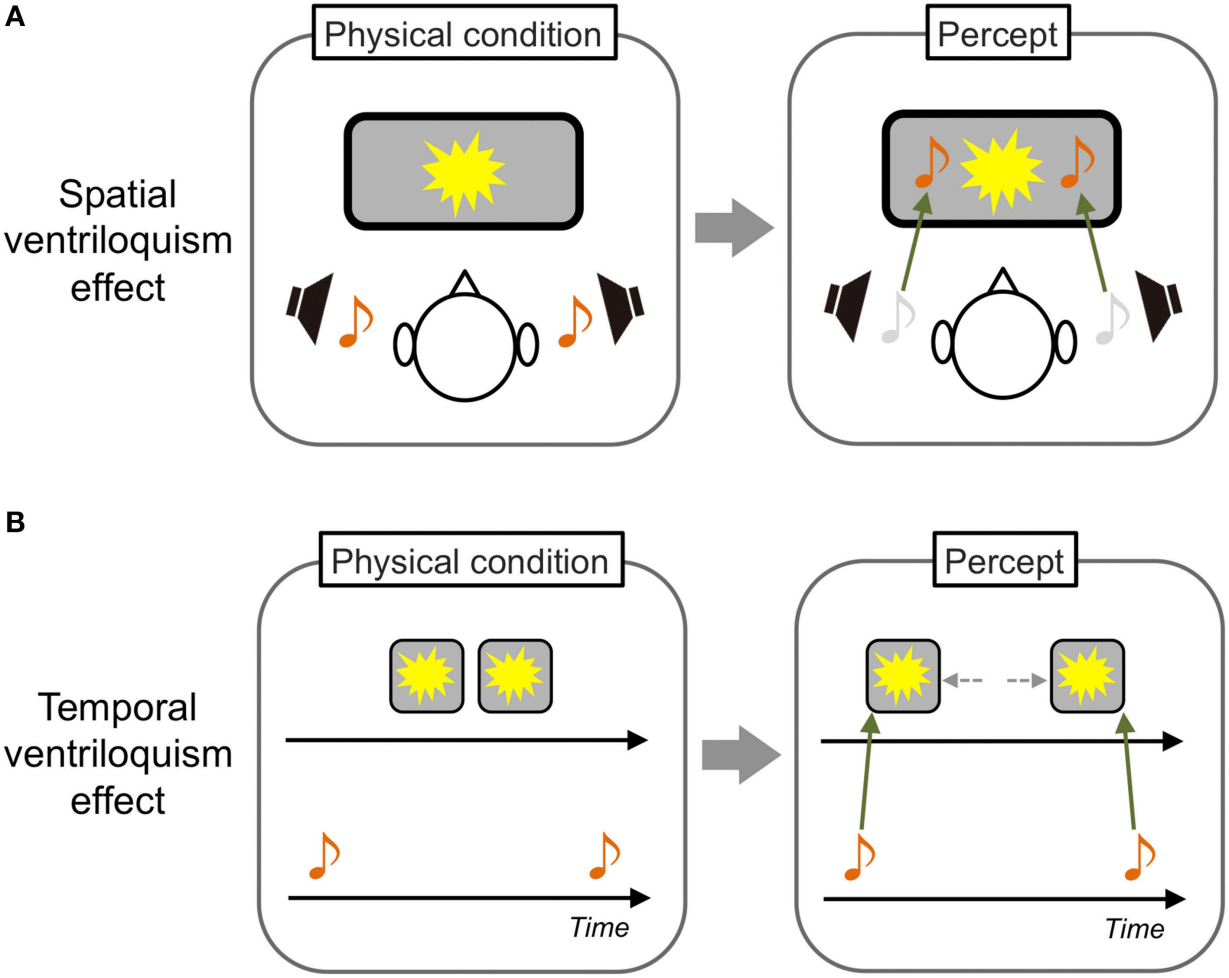
**Schematic illustrations of spatial and temporal ventriloquism effects**. **(A)** In a typical spatial ventriloquism effect, the spatial position of sound sources is perceived as that of visual sources. **(B)** In contrast, the temporal position of visual sources perceptually shifts to that of sound sources in a typical temporal ventriloquism effect.

One famous phenomenon in crossmodal interactions is the “spatial ventriloquism” effect. Typically, a visual event is presented in front of observers and a sound source related to the event is placed in a spatially discrepant position. In this situation, the sound is perceived as occurring at the position of the visual event (Howard and Templeton, 1966; Figure 1A). As such, the visual modality is known to be dominant over other sensory modalities in spatial processing. The reason could be simply that visual information inherently has the most precise resolution in the spatial domain (i.e., highest spatial resolution) among the sensory modalities (modality appropriateness hypothesis: Welch and Warren, 1980, 1986). However, the classical modality appropriateness hypothesis has been refined by evidence provided in this decade. Alais and Burr (2004b) demonstrated that sounds could have a dominant spatial ventriloquism effect on visual stimuli when the stimuli were presented as spatially ambiguous (see also Radeau and Bertelson, 1987). This obviously suggests that the manner of crossmodal interactions in the spatial domain is not just dependent on the unique property of each sensory modality. Rather, it would also be dependent on the relative certainty/reliability of the inputs (Ernst and Banks, 2002; Ernst and Bülthoff, 2004).

Crossmodal interactions in the spatial domain other than with vision have also been reported. For example, sound could affect tactile distance perception (Tajadura-Jiménez et al., 2012). Observers were exposed to a situation where sound locations were always moderately far away relative to the tapped position of their arm. After the exposure, the perceived distance of the tactile sensations on the observer's arm was greater than the actual stimulated positions by the sound presentations.

Crossmodal interactions have been also investigated not only in two-dimensional, but also in three-dimensional spaces (see Van der Stoep et al., 2015 for a review). For instance, Sugita and Suzuki (2003) reported that the timing of a perceived co-occurrence between light and sound was changed as a function of the distance between the observer and the light. This suggests that the brain compensates for temporal lag using viewing distance information, as if it “knows” the physical rule that the traveling velocity of light is faster than that of sound in space.

In traditional crossmodal interaction studies, it has been reported that interactions among crossmodal stimuli are most frequently observed when these stimuli are spatially congruent (spatial co-localization rule: Calvert et al., 2004; Stein, 2012). However, recent findings suggest that the “spatial co-localization rule” is not generally applicable for all crossmodal interactions for given phenomena and tasks (Spence, 2013 for a review). As mentioned previously, the understanding of crossmodal interaction in the spatial domain has been updated in recent years.

## Crossmodal interactions in temporal domain

In contrast to vision, audition and tactile sensation are known to be dominant in temporal processing. For example, an auditory driving effect has been reported; the perceived rate of visual flickers is modulated by the rate of concurrently presented sounds (Gebhard and Mowbray, 1959). Shams et al. (2000) reported that a single visual flash is perceived as double when sounds are concurrently presented twice. A similar temporal modulatory effect of auditory stimuli on tactile sensation was also reported (Bresciani et al., 2005). Furthermore, in a well-known “temporal ventriloquism” effect, judgments of the presentation order of two visual flashes were improved when two sounds were presented before and after the flashes. In contrast, judgments degraded when two sounds were interspersed between the flashes. These findings indicate that the perceived temporal position of the visual stimuli is captured by the sounds (Fendrich and Corballis, 2001; Morein-Zamir et al., 2003; Figure 1B). Regarding tactile sensation, researchers have reported that judgments of the presentation order of two tactile stimuli presented to observers' hands became worse when the hands were crossed. This indicates that tactile temporal perception could interact with proprioceptive information (Yamamoto and Kitazawa, 2001).

How is temporal information integrated across sensory modalities (vision, audition, and tactile sensation) and what kinds of rules exist for crossmodal temporal binding? Fujisaki and Nishida (2009) found that the maximum limits of temporal integration are superior for the combination of audition and tactile sensation over other combinations. This seems to indicate that unique temporal characteristics of each modality determine the upper temporal integration limits of each sensory pair. However, Fujisaki and Nishida (2010) also reported that similar differences in upper temporal integration limits could also be observed for different feature combinations in a single modality (i.e., color, luminance, and orientation in vision). According to a recent viewpoint, differences in temporal processing among sensory modalities are considered dependent on which processes are involved (e.g., bottom-up or top-down/attentional processes, “what,” “when,” or “where” processing; see Fujisaki et al., 2012 for a review).

## Crossmodal interactions in spatiotemporal domain

Thus far, we briefly overviewed crossmodal interactions in the spatial and temporal domains. The findings generally suggest that vision is superior to audition in spatial processing, but audition and tactile sensation is dominant over vision in temporal processing. These superiorities/dominances could be also changed in some cases depending on the stimuli's reliability and/or the processes involved. It should be noted, however, that the inputs in our surrounding environments are dynamic, so that spatial and temporal information are indivisible. On this point, studies on crossmodal interactions have focused on the spatiotemporal processing (namely, motion perception) of information from multiple senses. Next, we review past and recent findings regarding crossmodal interactions in motion perception (Table 1).

**Table 1:** **Summary of psychophysical research evidences of crossmodal motion perception**.

**Representative studies**	**Affecting modality**	**Motion information**	**Affected modality**	**Motion information**	**Effect domain**
**MODULATORY EFFECTS FROM VISION**					
Soto-Faraco et al., 2002	Vision	Yes	Audition	Yes	Motion
Konkle et al., 2009	Vision	Yes	Touch	Yes	Motion
**MODULATORY EFFECTS FROM SENSORY MODALITIES OTHER THAN VISION**					
Sekuler et al., 1997	Audition	No	Vision	Yes	Event
Getzmann, 2007	Audition	No	Vision	Yes	Time
Kim et al., 2012	Audition	Yes	Vision	Yes	Motion
Sanabria et al., 2005	Audition	Yes	Touch	Yes	Motion
Sanabria et al., 2005	Touch	Yes	Audition	Yes	Motion
Konkle et al., 2009	Touch	Yes	Vision	Yes	Motion
Kuang and Zhang, 2014^*^	Smell	No	Vision	Yes	Motion
**DRIVING EFFECTS**					
Kitagawa and Ichihara, 2002	Vision	Yes	Audition	No	Motion
Hidaka et al., 2009	Audition	Yes	Vision	No	Motion
Teramoto et al., 2010a^*^	Audition	No	Vision	Yes	Motion
Keetels and Stekelenburg, 2014	Motor action	Yes	Vision	No	Motion

* Effects appear after perceptual associative learning.

### Modulatory effects

**Figure 2:**
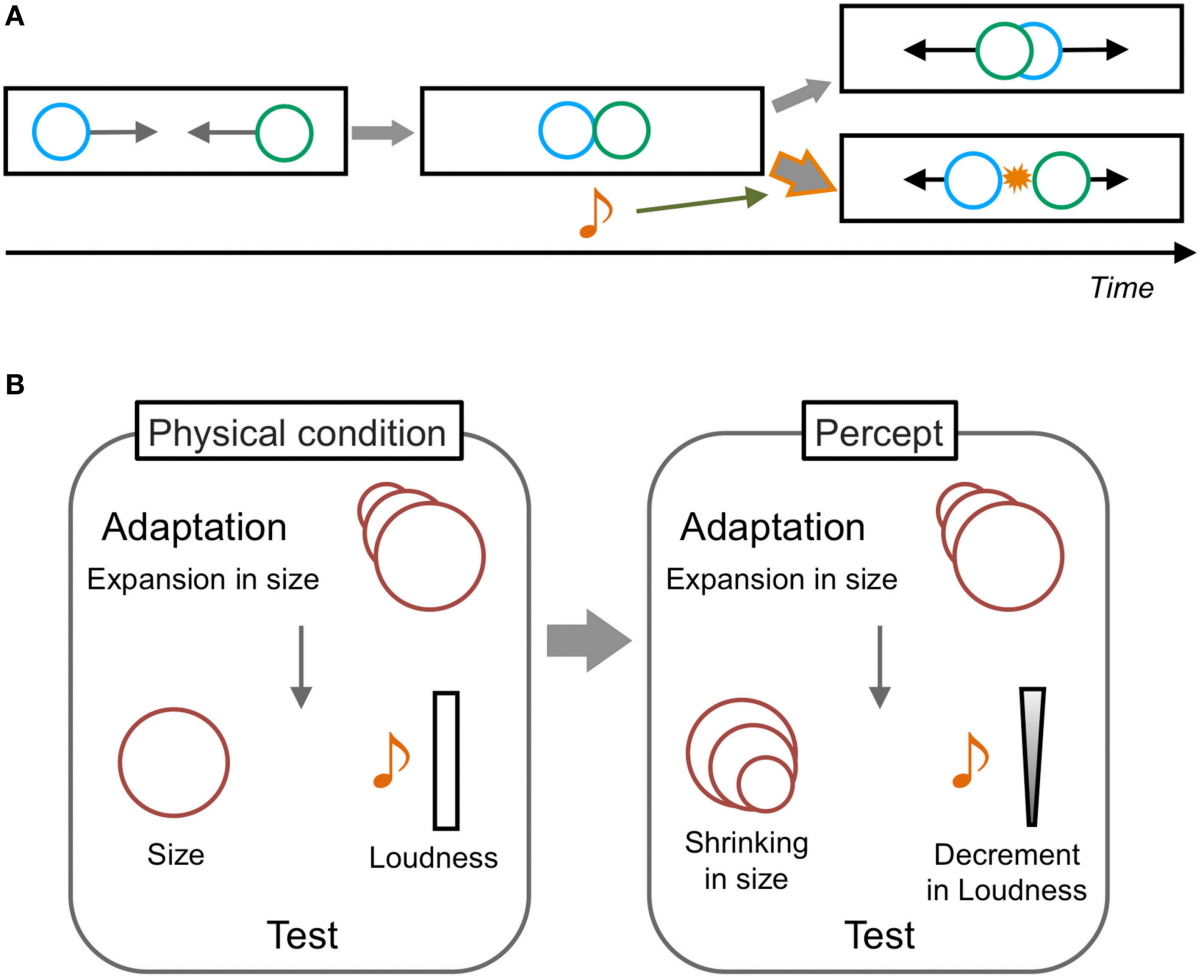
**Schematic illustrations of audiovisual illusions in motion perception**. **(A)** Stream-bounce illusion. Two visual stimuli moving toward each other at the same vertical location are perceived to overlap at the center and then to continue moving along their trajectories. Both streaming and bouncing percepts occur equally without auditory information. However, when a transient sound is presented at the timing of the visual coincidence, the bouncing perception becomes dominant. This is a typical auditory modulatory effect on visual motion perception. **(B)** Auditory aftereffects induced by visual adaptation. The adaptation to visual size changes (e.g., expansion) induces not only size change aftereffects for the visual test stimulus with constant size (shrinking), but also loudness change aftereffects for the auditory stimuli with constant loudness (decreasing in loudness). The involvement of motion processing could be assumed in this phenomenon because both auditory and visual stimuli are assumed to demonstrate motion in depth. The effect from audition to vision is not reported to occur.

As per the audio-visual interaction in motion perception, Sekuler et al. (1997) have reported a pioneering phenomenon called the “stream-bounce illusion.” In this illusion, two visual stimuli are presented as moving horizontally from opposite sides of a display toward each other at the same vertical location. The visual stimuli are perceived to overlap at the center of a display and then continue moving along their trajectories, and the perception of both streaming and bouncing can alternatively occur when only the visual stimuli are presented (Figure 2A). However, a transient sound induces dominant bouncing perception when the sound is presented at the overlap timing of the visual stimuli. This phenomenon is assumed to be purely based on audiovisual interactions (Watanabe and Shimojo, 2001). Sound has also been reported to change the perception of the onset timing of inducers of visual apparent motion; this consequently modulate the optimal presentation timings of visual apparent motion (Getzmann, 2007), the distance between apparently moving stimuli (Kawabe et al., 2008), and the perceived direction of an ambiguous visual apparent motion display (Freeman and Driver, 2008, but also see Roseboom et al., 2013). These findings indicate that sound can modulate the perception of visual motion through changes in the interpretation of a visual event or by temporal ventriloquism effects.

With regard to the effect of visual information in audiovisual spatiotemporal interactions, some studies have reported that visual stimuli changed the percept of a static sound as moving (e.g., Mateeff et al., 1985). Kitagawa and Ichihara (2002) demonstrated that the co-presentation of visual size changes (increasing or decreasing the size of visual images) with an auditory loudness change in a congruent direction could enhance the auditory loudness aftereffects (Figure 2B). Moreover, the adaptation to visual size changes alone could induce the auditory loudness change of a steady sound in the opposite direction of the visual adapting stimuli. In this situation, both auditory and visual stimuli are assumed to signify motion in depth and the aftereffect is considered to occur purely at perceptual levels. These findings thus suggest that visual motion information could directly affect or induce auditory motion perceptions. In contrast, in their study, the auditory loudness change did not have any modulatory or inducing effect on visual aftereffects.

**Figure 3:**
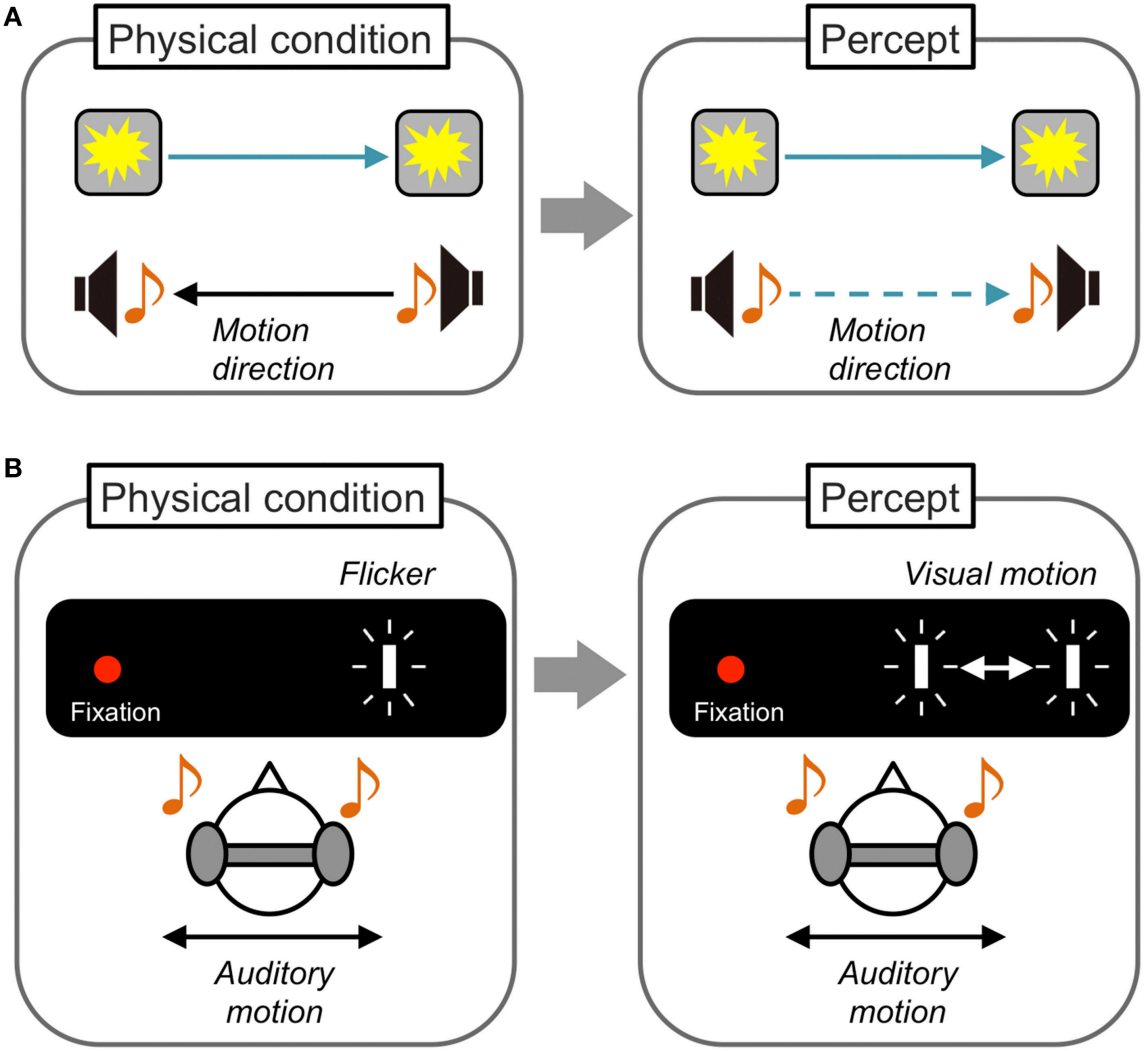
**Schematic illustrations of dynamic visual capture and sound-induced visual motion. (A)** Dynamic visual capture. When the motion direction of apparent motion is incongruent between visual and auditory stimuli, the direction of auditory apparent motion is perceived as congruent with that of visual apparent motion. The opposite effect is reported less often. **(B)** Sound induced visual motion. Visual flashes presented at a fixed position are perceived as moving horizontally when auditory stimuli are presented in horizontal motion, especially at larger eccentricities. This is the first demonstration of an auditory driving effect on visual motion perception.

While these audiovisual interactions in motion perception have been mainly demonstrated between moving and static/constant stimuli, Soto-Faraco and his colleagues have reported crossmodal interactions when auditory and visual stimuli were both dynamic (Figure 3A). Soto-Faraco et al. (2002) presented auditory stimuli through speakers set at horizontally separated positions so that auditory apparent motion was perceived. Visual stimuli were concurrently presented from LEDs attached to speakers to induce visual apparent motion. Observers correctly reported the motion direction (left/right) of auditory apparent motion when its direction was consistent with that of visual apparent motion. However, when the motion direction was inconsistent with the auditory and visual stimuli, the observers tended to misperceive the direction of auditory motion to be consistent with the visual direction. Together with the findings in several control experiments manipulating the spatial and temporal relationship between auditory and visual stimuli, they concluded that this visual capture effect on auditory motion reflects direct crossmodal interactions in motion perception. Based on detailed investigations including testing of sensory pairs other than audiovisual stimuli (Soto-Faraco et al., 2003, 2004a,b, 2005; Sanabria et al., 2005), Soto-Faraco et al. have concluded that common perceptual mechanisms and shared neural substrates for different sensory information exist in motion perception. They have also suggested that vision is superior to other sensory information in crossmodal interactions in motion perception, because sounds did not have such capturing effects on vision.

### Driving effects

The perceived direction of visual stimuli moving in opposing directions in the vertical dimension could be biased by sounds with a change in pitch (i.e., rising pitch induced upward visual motion perception and falling pitch induced downward visual motion perception; Maeda et al., 2004). The sensitivity to detect horizontal visual motion was also improved by sounds moving in a consistent direction (Kim et al., 2012). Consistent with the aforementioned auditory effect on the interpretation of visual events and temporal ventriloquism effects on visual stimuli, these findings clearly demonstrate the modulatory effect of sound on visual motion perception (see Blake et al., 2004 for tactile modulatory effect on visual motion perception). In contrast, little or no auditory driving or inducing effects on visual motion perception have been demonstrated (Meyer and Wuerger, 2001; Wuerger et al., 2003; Alais and Burr, 2004a; Soto-Faraco et al., 2004b; but see Kitagawa and Ichihara, 2002 for visual driving effect on audition). In these studies, visual stimuli were presented clearly at a foveal or parafoveal position so that the percept of visual motion was salient. However, visual dominance over audition in the spatial domain collapsed when the visibility or reliability of visual inputs were degraded (Alais and Burr, 2004b). We can therefore predict that auditory information could have capturing or inducing effects on visual motion perception in this situation.

From this viewpoint, Hidaka and his colleagues have demonstrated an auditory driving effect on visual motion perception (Figure 3B). In their experiment (Hidaka et al., 2009), auditory apparent motion was presented through headphones and a blinking visual target at a fixed location was presented either in foveal, parafoveal, or perifoveal positions. They found that the static visual target tended to be perceived as moving when it was presented at the perifoveal position (>10°). This auditory driving effect on visual motion perception was reported not only for horizontal, but also for vertical auditory motion (Teramoto et al., 2010b). Furthermore, Hidaka et al. (2011b) found that auditory continuous motion information induced visual motion perception for a static visual target. In addition, the auditory continuous motion determined the perceived direction of an ambiguous visual global motion display in which motion information was extracted from the integration of multiple local motion signals (Williams and Sekuler, 1984). These findings indicate that the auditory driving effect could be dissociated from the attentional spatial capture effect (Spence and Driver, 1997) or auditory spatial bias effect on visual targets (Radeau and Bertelson, 1987; Alais and Burr, 2004b). Rather, the effect is considered to be purely based on perceptual motion processing.

Recent studies have shown that similar effects are observed beyond the auditory-visual domain. Fracasso et al. (2013) reported that the auditory induced illusory visual motion triggered a visuo-motor response (eye movement) similar to actual visual motion. Keetels and Stekelenburg (2014) found that motor induced motion information (finger movements) also induce visual motion perception for a static visual target. Furthermore, it was recently reported that motion aftereffects could mutually transfer between visual and tactile modalities (Konkle et al., 2009).

These findings could extend the suggestions originally proposed by Soto-Faraco et al. Different sensory information flexibly and adequately cooperates *with each other* based on the reliability and saliency of information under common perceptual mechanisms and shared neural substrates in motion perception.

### Effects of associative learning

How are common perceptual and neural mechanisms established in the brain across sensory modalities in motion perception? By considering the fact that each sensory organ has unique properties in perceptual processing, we may learn the manner in which the inputs of different sensory modalities should be associated or integrated after birth. The influential cue for the integration/association between sensory modalities is considered to be spatiotemporal consistency/proximity (Calvert et al., 2004). Research on crossmodal perceptual learning reported that repeated/redundant presentations of paired moving visual and auditory stimuli induced the facilitation of visual motion discrimination (Seitz et al., 2006; Kim et al., 2008). In addition, reliability of crossmodal inputs was also reported to affect the establishment of crossmodal associations (Ernst et al., 2000). Based on these findings, the establishment of crossmodal associations has been considered in the context of a maximum likelihood estimation model (Ernst and Banks, 2002). However, this type of model lacks the viewpoint of how we know to utilize spatiotemporal information and/or reliability as influential cues to decide whether crossmodal inputs should be integrated or segregated. Recently, Bayesian models/frameworks have approached this problem (Ernst, 2007). Körding et al. (2007) designed a model implementing the prior knowledge of whether auditory and visual stimuli should be integrated based on spatial proximity of these stimuli. They demonstrated that their model predicted behavioral performances of audio-visual spatial ventriloquism effects well (see also Shams and Beierholm, 2010 for a review). Further, not only spatiotemporal proximity but also correlative relationships of crossmodal inputs can play a key role in the determination of integration and segregation of these inputs (Parise et al., 2012, 2013). These findings indicate that any combination of crossmodal stimuli is possible to be integrated if prior knowledge or experiences established that they are associable (see also van Dam et al., 2014 for a review). In fact, new associations/relationships could be learned across arbitrary static/constant crossmodal inputs, even by adults (Fujisaki et al., 2004; Ernst, 2007; Seitz et al., 2007). Thus, we could predict that arbitrary crossmodal associations could be established in motion perception as well.

**Figure 4:**
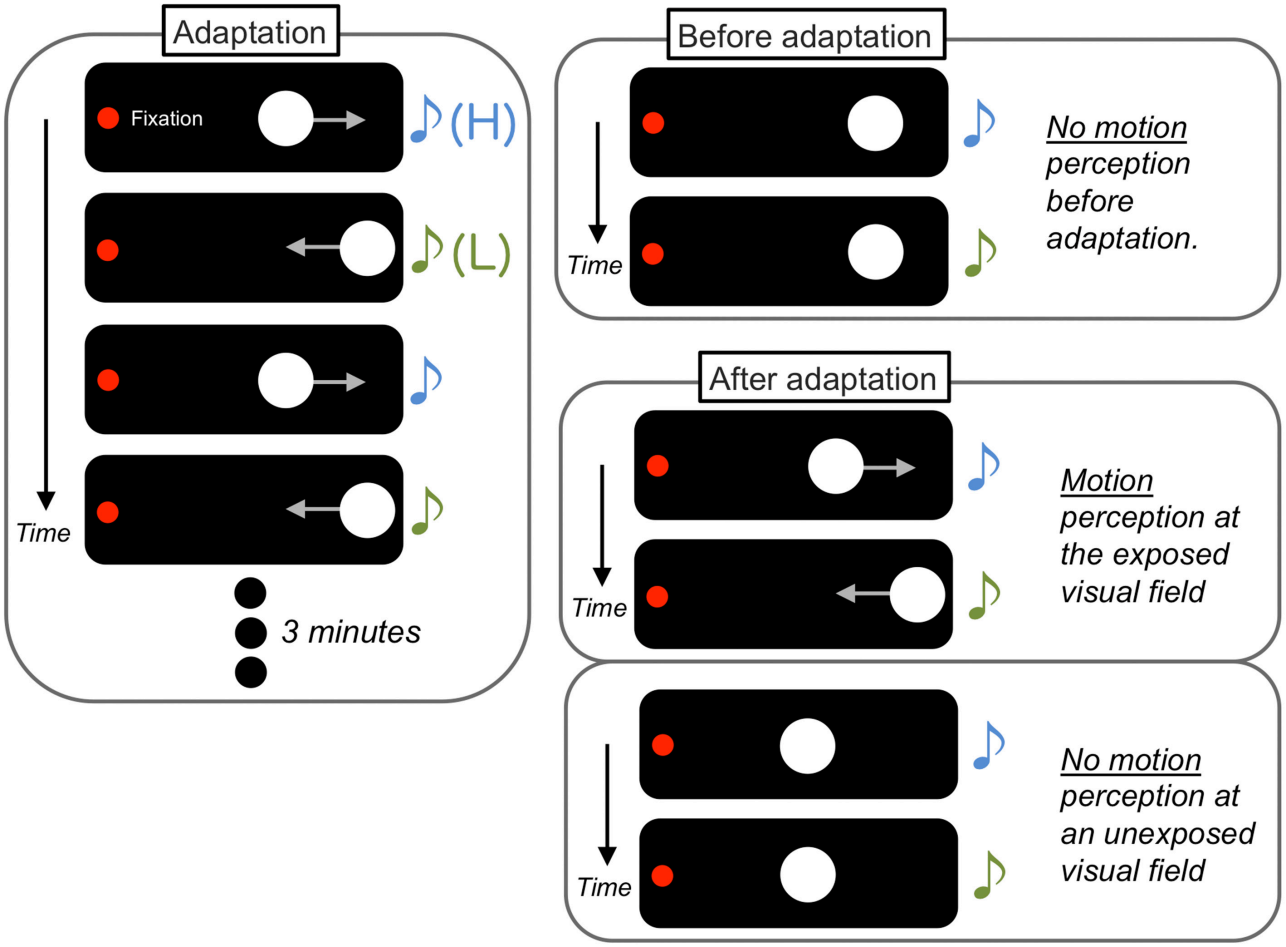
**Schematic illustrations of perceptual associative learning between a sound sequence and visual apparent motion**. After a 3 min exposure (adaptation) to a paired presentation of sound with arbitrary pitch changes [higher (H) to lower (L)] and visual horizontal apparent motion (leftward motion), the sounds began to induce visual apparent motion in the previously exposed manner, specifically at the exposed visual field.

Teramoto et al. (2010a) demonstrated that sounds without spatial information become a driver for visual motion perception after associative learning (Figure 4). In their experiments, two visual flashes were presented as visual apparent motion stimuli in a horizontal direction. The onset of each visual stimulus was accompanied by an auditory stimulus in one of two arbitrary pitches (higher (H) or lower (L)). Before a 3 min exposure to a paired presentation of the visual and auditory stimuli (e.g., leftward motion and H to L pitch change and rightward motion and L to H pitch change), the sounds did not affect the percept of visual apparent motion. In contrast, after the exposure, the sounds induced visual apparent motion in the exposed manner (in this case, the H to L pitch change induced leftward motion perception and vice versa). These association effects did not appear when the inter-stimulus interval of the visual stimuli was too long to be perceived as apparent motion during the exposure. In addition, the association effect was also found for the pairing of auditory pitch changes and directional information in a visual global motion display (Michel and Jacobs, 2007; Hidaka et al., 2011a). Kafaligonul and Oluk (2015) have also reported that the exposure to auditory pitch changes and higher-order visual motion induced the association effect on both lower- and higher-order visual motion perception. In contrast, the exposure to auditory pitch changes and lower-order visual motion induced the association effect only for lower-order visual motion perception. These findings indicate that motion processing plays a key factor in the establishment of crossmodal associations in motion perception.

Kobayashi et al. (2012a) presented auditory frequency changes that were physically different but perceptually indiscriminable in their experiments. They demonstrated that the undetectable frequency differences were unconsciously extracted and utilized for establishing associations with visual apparent motion. Furthermore, the effect did not occur when the stimulated eyes differed between the exposure and test sessions. Kobayashi et al. (2012b) further reported that the association effects produced sharp selectivity in auditory processing. After exposure to visual apparent motion and a specific frequency change of tones (e.g., 400 and 2100 Hz), the tones that differed from the previously associated frequencies by 0.25 octave (476–2496 or 566–2970 Hz in this case) did not have any effect on visual motion perception in the subsequent test session. Similarly, when the sounds were presented in the right ear of the observers during the exposure, sounds presented in the left ear did not affect visual motion perception. Similar selectivity was reported for the visual domain. The effect of the exposure to auditory pitch changes and the visual moving stimuli (e.g., at 10° to the right side of the visual field) were not observed at the contralateral side of the visual display (left side in this case), or even at the ipsilateral side with small deviations (e.g., 5° to the right side of the visual field; Teramoto et al., 2010a; Hidaka et al., 2011a). Similar eye field selectivity was found in the association effects from visual to auditory stimuli (i.e., visual motion information induced changes in the percept of auditory pitches; Teramoto et al., 2013). These selective aspects suggest that, under the establishment of associations in crossmodal spatiotemporal processing, some lower level of processing could be involved such as subthreshold processing, monaural/monocular processing, and the processing of frequency band and receptive fields.

These aforementioned perceptual association paradigms in crossmodal motion perception have been utilized for further investigations. The existence of crossmodal correspondences is well-known. For example, higher/lower pitch information could induce upper/lower impressions in space and changes in response (e.g., reaction time; Bernstein and Edelstein, 1971; see also Spence, 2011 for a review). Hidaka et al. (2013) investigated whether this pitch-space correspondence could have a perceptual effect on motion perception. They found that, different from the spatial alternation of sound locations in a vertical direction, the alternation of pitch information (higher and lower) did not induce vertical visual apparent motion perception. In contrast, after the association was established between the alternation of pitch information and visual vertical apparent motion, the pitch changes affected visual motion perception. A notable point is that the association effects appeared to demonstrate pitch-space correspondence rules. The upward and downward directions of visual apparent motion were triggered by lower-to-higher and higher-to-lower pitch changes, respectively, regardless of the manner of the association between the pitch change (lower to higher or higher to lower) and the upward/downward visual motion in the exposure phase. The authors speculated that the associative exposure could activate the existing representations of pitch-space correspondence to induce their crossmodal effects on motion perception. Kuang and Zhang (2014) investigated whether a sensory combination other than audition and vision could produce association effects. They presented changes in smells (banana and fennel) with a visual global motion display. After exposure to these stimuli, the smells affected the perceived direction of the visual global motion display. This result suggests that crossmodal associative learning in spatiotemporal processing is not limited to audio-visual domains, but could generally occur among a variety of sensory pairs.

Recent findings clearly suggest that new perceptual associations could be established between arbitrary inputs through crossmodal spatiotemporal processing. After associations are formed, each sensory input affects the percept of the other one as if “replaying” the associated relationship. The association effects are assumed not to be limited to particular sensory combinations, but have sharp selectivity in each sensory modality. These findings suggest that perceptual associative learning is one of the most plausible underlying mechanisms to establish common perceptual and neural mechanisms in crossmodal spatiotemporal processing.

### Functional brain characteristics in crossmodal spatiotemporal processing

Neural substrates of crossmodal interactions have been investigated using neurophysiological and brain imaging techniques in animals and humans. Researchers have shown that multisensory inputs could activate both subcortical (e.g., superior colliculus, pulvinar nucleus, and putamen) and cortical (e.g., sensory association areas in the temporal, parietal, and frontal) regions, and even brain areas that have been considered as primary sensory areas (e.g., visual and auditory areas; see Calvert, 2001; Driver and Noesselt, 2008; Murray and Wallace, 2011 for review).

Some researchers have investigated the neural mechanisms for motion processing in crossmodal interactions by using brain-imaging techniques. Lewis et al. (2000) presented visual and auditory motion stimuli independently and then investigated the overlapped and non-overlapped activation areas for those inputs. These motion stimuli commonly activated the lateral parietal, lateral frontal, anterior midline, and anterior insula areas. In contrast, visual and auditory stimulation independently activated the primary visual and V5/MT areas and the auditory primary areas as well as the surrounding areas including the periarcuate cortex, respectively. Interestingly, the inferior parietal lobule, dorsal occipital cortex, and the cortex overlapping hMT+ were activated by visual motion but suppressed by auditory motion. Auditory motion information strongly activated the superior temporal sulcus. In addition, during a speeded discrimination task for these motion stimuli, the intraparietal sulcus, anterior midline, and anterior insula were activated. Researchers have also reported that visual, auditory, and tactile motion information, which were independently presented, activated identical sensory association areas, such as the intraparietal sulcus, as well as the lateral inferior postcentral cortex and the premotor cortex (Bremmer et al., 2001; see also Grefkes and Fink, 2005).

Furthermore, Baumann and Greenlee (2007) found that the brain areas related to crossmodal integration (e.g., the superior parietal lobule, the superior temporal gyrus, the intraparietal sulcus, and the supra marginal gyrus) were activated when visual random-dot motion display and auditory motion stimuli were presented in a congruent direction. However, activation in the V5/MT area was not observed. The authors speculated that this might be due to relatively weak visual motion stimulation. In contrast, Alink et al. (2008) reported that the activation in the V5/MT area became higher when the visual and auditory motion signals were presented as coherent rather than as incoherent. Interestingly, auditory motion information alone also activated the V5/MT area (see also Alink et al., 2012). Similarly, the areas identified to respond to auditory motion (auditory motion complex: AMC) were also activated by visual motion information. In addition, when visual motion information perceptually captured auditory information (Soto-Faraco et al., 2002), activation was enhanced in the V5/MT area, while AMC activation decreased.

Scheef et al. (2009) used a complex situation in which a visual motion signal containing biological meaning (i.e., a human jumping) and sounds consistent with visual motion (implying the jumping) were presented. They reported that activation in the V5/MT area, as well as in the superior temporal sulcus, the intraparietal complex, and the prefrontal regions, was enhanced by the sounds. Studies have also indicated that tactile motion information could activate the V5/MT area, as well as the somatosensory areas, similar to a visual motion signal or an interaction with visual information (Hagen et al., 2002; Blake et al., 2004; van Kemenade et al., 2014).

There have also been several electrophysiological studies regarding crossmodal interactions in motion perception. For instance, Stekelenburg and Vroomen (2009) focused on early event-related mismatch negativity (MMN) components (around 200 ms). They reported that MMN induced by changes in the auditory motion direction diminished when the visual capture effect on auditory motion occurred. Since MMN is assumed to reflect automatic, pre-attentive processes, these findings indicate the involvement of perceptual processes in crossmodal motion perception (see also Beer and Röder, 2005; Zvyagintsev et al., 2009). Moreover, congruent audio-visual (Gleiss and Kayser, 2014) and visuo-tactile (Krebber et al., 2015) motion information enhanced alpha-band and gamma-band activities in each primary sensory area. This suggests that both top-down and bottom-up processes underlie the integration of crossmodal motion information. While these studies mainly used motion stimuli in a two-dimensional plane, recent studies also demonstrate the involvement of early sensory areas to process audio-visual crossmodal stimuli simulating motion in depth, especially looming stimuli (Romei et al., 2009; Cappe et al., 2012; Ogawa and amd Macaluso, 2013).

Taken together, crossmodal motion information could activate from higher sensory association areas (e.g., the intraparietal and superior temporal sulcus) to a relatively lower motion processing areas (e.g., V5/MT area) and primary sensory areas related to motion processing. The activation patterns in these areas are also assumed to be variable depending on the congruency of motion signals, the types of stimuli, and the experimental paradigm.

### Possible linkages between perceptual and neural aspects

Thus far, we overviewed recent literatures regarding perceptual aspects and functional brain characteristics related to crossmodal spatiotemporal processing. Here, we discuss possible linkages between these aspects.

**Figure 5:**
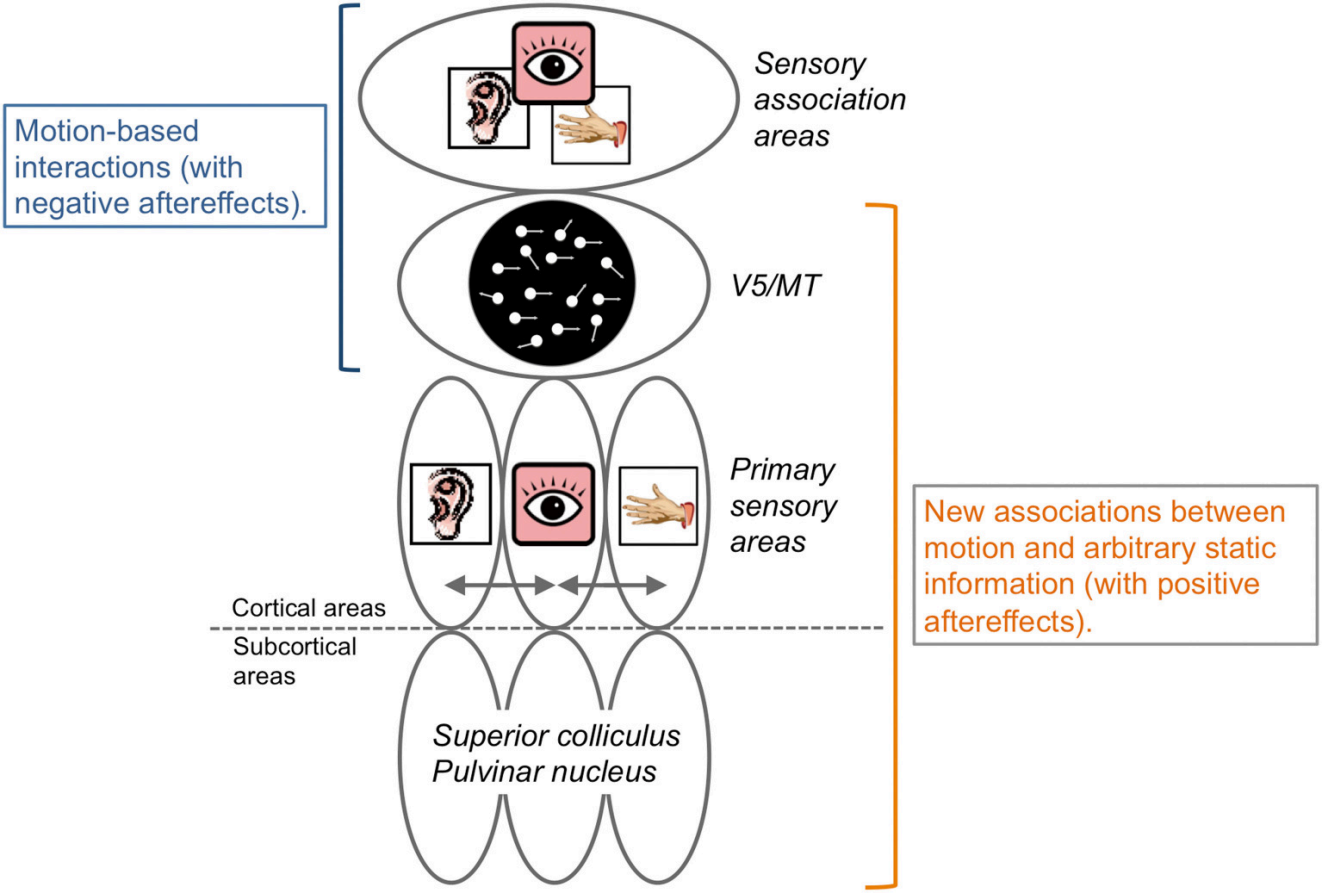
**Schematic illustrations of neural bases of crossmodal motion perception**. Interactions of motion information across sensory modalities could occur in V5/MT and association areas and induce negative aftereffects. In contrast, it could be assumed that some lower levels of brain representation and processing areas, including the subcortical, primary sensory, and V5/MT areas, play key roles for the establishment of new neural representations regarding the associations of motion information and arbitrary information without motion signals with positive aftereffects. See main text for details.

Some psychophysical studies have shown that motion aftereffects have occurred across sensory modalities (Kitagawa and Ichihara, 2002; Konkle et al., 2009). Specifically, the aftereffect was *negative* (e.g., adaptation to upward motion subsequently induced downward motion perception for static stimuli). Visual *negative* motion aftereffects are assumed to occur due to the inhibition of neurons selective for the adapted motion direction and the activation/enhancement of neurons with selectivity opposite to the adapted direction (Anstis et al., 1998). In this case, we could assume that motion directional neurons in V5/MT and the sensory association areas (e.g., the superior temporal sulcus; Bruce et al., 1981) mediated the interplay of motion processing across sensory modalities, as shown in the brain imaging studies (Lewis et al., 2000; Bremmer et al., 2001; Grefkes and Fink, 2005; Baumann and Greenlee, 2007; Alink et al., 2008; Figure 5).

On the other hand, studies on crossmodal perceptual associative learning have consistently demonstrated *positive* aftereffects (Michel and Jacobs, 2007; Teramoto et al., 2010a; Hidaka et al., 2011a; Kuang and Zhang, 2014; Kafaligonul and Oluk, 2015). In these studies, visual motion stimuli were presented with sounds or smells not containing any motion stimuli. In a crossmodal *negative* motion aftereffect, motion information is clearly presented in multiple modalities so that existing neural representations responsible for motion processing are considered to be involved. In contrast, during crossmodal perceptual associative learning, we could assume that new neural representations are established between motion information and arbitrary information without a motion signal (c.f. Haijiang et al., 2006). After the association is formed, the arbitrary information simply works as a predictive cue for motion perception to the paired stimuli to induce a *positive* aftereffect. In line with this idea, Schlack and Albright (2007) reported that, after associations were established between the orientation information of static visual arrows and motion directions, neurons in the MT area of rhesus monkeys became selective for the orientation information of the static arrows. Moreover, the behavioral results showed that audiovisual perceptual association effects in motion perception have visual field selectivity ranging within 5° at 5–10° of eccentricity (Teramoto et al., 2010a; Hidaka et al., 2011a). This spatial selectivity aspect almost matches the V5/MT neurons receptive field's size (e.g., Felleman and Kaas, 1984). We could assume that the area involved in motion processing (V5/MT area) would be the potential brain region where new neural representations for crossmodal motion processing are formed in crossmodal *positive* motion aftereffects.

As discussed above, functional brain research regarding crossmodal spatiotemporal processing focused on cortical activities with higher sensory association areas to motion processing areas. However, in spatial or temporal processing, multisensory inputs were reported to activate both subcortical and lower cortical regions. Additionally, audiovisual perceptual association effects in motion perception demonstrated sharp selectivity in both visual (eye selectivity; Kobayashi et al., 2012a) and auditory (ear and frequency selectivity; Kobayashi et al., 2012b) domains. These would indicate that lower cortical regions such as primary sensory areas and/or subcortical regions could play key roles for crossmodal motion processing.

Consistent with the suggestions from many crossmodal studies, lower and higher brain regions and bottom-up and top-down processing would be mutually and closely involved in crossmodal interactions in spatiotemporal processing.

## Concluding remarks

In this review, we encapsulated the recent evidence regarding crossmodal interactions in spatiotemporal processing (i.e., motion perception). The traditional view in crossmodal studies has regarded the dominant effects of vision over the other sensory modalities (i.e., visual capture) in spatiotemporal processing. Sensory information, other than from vision (e.g., sound), had been assumed to have only modulatory effects on crossmodal motion perception. However, recent findings clearly demonstrate that sound and motor action could have a driving effect on visual motion perception, specifically when the visibility of the visual stimuli was degraded. Studies regarding perceptual associative learning have further reported that an association could be established between sounds without spatial information and visual motion information by a 3 min exposure. Then, the sounds acquired a driving effect on visual motion perception. Other sensory information (smell) was also reported to have similar driving effects on visual motion perception. Crossmodal interactions studies at neural levels demonstrate that activation in lower and higher cortical brain regions, including the area related to visual motion processing, is commonly modulated by crossmodal motion information.

These findings clearly suggest that multimodal information could mutually interact in spatiotemporal processing and that common perceptual and neural underlying mechanisms for crossmodal spatiotemporal processing would exist. Importantly, it has been also demonstrated that crossmodal interactions in motion perception flexibly and adequately occur, based on the reliability and saliency of information in spatiotemporal domain. The brain activation patterns related to crossmodal motion perception are also considered to be variable depending on the congruency of motion signals. These characteristics would be concordant with recent Bayesian models/frameworks in crossmodal integrations assuming that prior knowledge/experience whether crossmodal inputs should be integrated or segregated play key roles (Shams and Beierholm, 2010; van Dam et al., 2014). The perceptual associative learning effects also indicate that arbitrary, unrelated crossmodal spatiotemporal information could interact if prior knowledge/experiences of integration are formed between them. These indicate that perceptual associative learning is one of the most plausible underlying mechanisms to establish common perceptual and neural representations of crossmodal spatiotemporal processing in the brain.

Several research questions remain to be addressed in future studies. For example, studies regarding crossmodal spatiotemporal processing have mainly investigated the interactions between vision and other sensory modalities. Visual information has been considered the most influential input regarding motion perception. Therefore, past findings are assumed to inevitably include the products of visual processing and related neural activities (e.g., the involvement of the V5/MT area). Of course, our perceptual systems can also receive motion information from the auditory and tactile sensory modalities that affected visual motion perception. Some studies have reported that auditory or tactile motion can be perceived against bilateral lesions of the lateral occipital cortex, including V5/MT and/or the posterior parietal cortex, while this induced visual motion blindness (Zihl et al., 1983, 1991; Rizzo et al., 1995). Moreover, spatiotemporal information not only exists in the external world. Internal spatiotemporal information, namely vestibular sensation such as head movement or self-motion, are also present and they interact and coordinate with other sensory ones (see Angelaki and Cullen, 2008 for a review). Thus, the generalization and validity of the existing evidence regarding crossmodal spatiotemporal processing should be confirmed by focusing on crossmodal interactions excluding vision.

Detailed investigation regarding the process of establishing neural substrates through perceptual associative learning is also necessary. For example, when and where do new neural substrates appear in the brain during perceptual associative learning? Which brain areas begin to activate and how does the amount of neural activity change over time? Does the perceptual aftereffect shift from positive to negative along with the development of forming neural representations? Answers to these questions could contribute to an understanding of the manner in which the brain acquires perceptual and neural bases for crossmodal interactions in spatiotemporal processing.

We should also focus on the possible effects that occur from spatiotemporal processes to spatial and temporal processes, while previous studies have mainly investigated the opposite. For example, in the visual domain, motion information changes the percept of surrounding space (Whitney and Cavanagh, 2000). In crossmodal studies, vestibular motion information, such as head movement (Leung et al., 2008) and forward self-motion (Teramoto et al., 2012), could distort the percept of auditory space. Further investigation of this aspect could contribute to e comprehensive understanding of the influence of crossmodal interactions in spatiotemporal, spatial, and temporal processing.

## Author contributions

SH and WT are involved in literature search. SH, WT, and YS are involved in the interpretations of findings and the writing of the paper including figures and tables.

### Conflict of interest statement

The authors declare that the research was conducted in the absence of any commercial or financial relationships that could be construed as a potential conflict of interest.

## References

[B1] Alais D. Burr D. (2004a). No direction-specific bimodal facilitation for audiovisual motion detection. Brain Res. Cogn. Brain Res. 19, 185–194. 10.1016/j.cogbrainres.2003.11.011 15019714

[B2] Alais D. Burr D. (2004b). The ventriloquist effect results from near-optimal bimodal integration. Curr. Biol. 14, 257–262. 10.1016/j.cub.2004.01.029 14761661

[B3] Alink A. Euler F. Kriegeskorte N. Singer W. Kohler A. (2012). Auditory motion direction encoding in auditory cortex and high-level visual cortex. Hum. Brain Mapp. 33, 969–978. 10.1002/hbm.21263 21692141 PMC6870293

[B4] Alink A. Singer W. Muckli L. (2008). Capture of auditory motion by vision is represented by an activation shift from auditory to visual motion cortex. J. Neurosci. 28, 2690–2697. 10.1523/JNEUROSCI.2980-07.2008 18337398 PMC6670665

[B5] Angelaki D. E. Cullen K. E. (2008). Vestibular system: the many facets of a multimodal sense. Annu. Rev. Neurosci. 31, 125–150. 10.1146/annurev.neuro.31.060407.125555 18338968

[B6] Anstis S. Verstraten F. A. Mather G. (1998). The motion aftereffect. Trends Cogn. Sci. (Regul. Ed). 2, 111–117. 10.1016/S1364-6613(98)01142-5 21227087

[B7] Baumann O. Greenlee M. W. (2007). Neural correlates of coherent audiovisual motion perception. Cereb. Cortex 17, 1433–1443. 10.1093/cercor/bhl055 16928890

[B8] Beer A. L. Röder B. (2005). Attending to visual or auditory motion affects perception within and across modalities: an event-related potential study. Eur. J. Neurosci. 21, 1116–1130. 10.1111/j.1460-9568.2005.03927.x 15787717

[B9] Bernstein I. H. Edelstein B. A. (1971). Effects of some variations in auditory input upon visual choice reaction time. J. Exp. Psychol. 87, 241–247. 10.1037/h0030524 5542226

[B10] Blake R. Sobel K. V. James T. W. (2004). Neural synergy between kinetic vision and touch. Psychol. Sci. 15, 397–402. 10.1111/j.0956-7976.2004.00691.x 15147493

[B11] Bremmer F. Schlack A. Shah N. J. Zafiris O. Kubischik M. Hoffmann K. P.. (2001). Polymodal motion processing in posterior parietal and premotor cortex: a human fMRI study strongly implies equivalencies between humans and monkeys. Neuron 29, 287–296. 10.1016/S0896-6273(01)00198-2 11182099

[B12] Bresciani J. P. Ernst M. O. Drewing K. Bouyer G. Maury V. Kheddar A. (2005). Feeling what you hear: auditory signals can modulate tactile tap perception. Exp. Brain Res. 162, 172–180. 10.1007/s00221-004-2128-2 15791465

[B13] Bruce C. Desimone R. Gross C. G. (1981). Visual properties of neurons in a polysensory area in superior temporal sulcus of the macaque. J. Neurophys. 46, 369–384. 6267219 10.1152/jn.1981.46.2.369

[B14] Calvert G. A. (2001). Crossmodal processing in the human brain: insights from functional neuroimaging studies. Cereb. Cortex 11, 1110–1123. 10.1093/cercor/11.12.1110 11709482

[B15] Calvert G. A. Spence C. Stein B. E. (eds.). (2004). The Handbook of Multisensory Processing. Cambridge: MIT Press.

[B16] Cappe C. Thelen A. Romei V. Thut G. Murray M. M. (2012). Looming signals reveal synergistic principles of multisensory integration. J. Neurosci. 32, 1171–1182. 10.1523/JNEUROSCI.5517-11.2012 22279203 PMC6796264

[B17] Driver J. Noesselt T. (2008). Multisensory interplay reveals crossmodal influences on ‘sensory-specific’ brain regions, neural responses, and judgments. Neuron 57, 11–23. 10.1016/j.neuron.2007.12.013 18184561 PMC2427054

[B18] Driver J. Spence C. (1998). Attention and the crossmodal construction of space. Trends Cogn. Sci. (Regul. Ed). 2, 254–262. 10.1016/S1364-6613(98)01188-7 21244924

[B19] Ernst M. O. (2007). Learning to integrate arbitrary signals from vision and touch. J. Vis. 7:7. 10.1167/7.5.7 18217847

[B20] Ernst M. O. Banks M. S. (2002). Humans integrate visual and haptic information in a statistically optimal fashion. Nature 415, 429–433. 10.1038/415429a 11807554

[B21] Ernst M. O. Banks M. S. Bülthoff H. H. (2000). Touch can change visual slant perception. Nat. Neurosci. 3, 69–73. 10.1038/71140 10607397

[B22] Ernst M. O. Bülthoff H. H. (2004). Merging the senses into a robust percept. Trends Cogn. Sci. (Regul. Ed). 8, 162–169. 10.1016/j.tics.2004.02.002 15050512

[B23] Felleman D. J. Kaas J. H. (1984). Receptive-field properties of neurons in middle temporal visual area (MT) of owl monkeys. J. Neurophys. 52, 488–513. 6481441 10.1152/jn.1984.52.3.488

[B24] Fendrich R. Corballis P. M. (2001). The temporal cross-capture of audition and vision. Percept. Psychophys. 63, 719–725. 10.3758/BF03194432 11436740

[B25] Fracasso A. Targher S. Zampini M. Melcher D. (2013). Fooling the eyes: the influence of a sound-induced visual motion illusion on eye movements. PloS ONE 8:e62131. 10.1371/journal.pone.0062131 23637981 PMC3637444

[B26] Freeman E. Driver J. (2008). Direction of visual apparent motion driven solely by timing of a static sound. Curr. Biol. 18, 1262–1266. 10.1016/j.cub.2008.07.066 18718760 PMC2882789

[B27] Fujisaki W. Kitazawa S. Nishida S. (2012). Multisensory timing, in The New Handbook of Multisensory Processing, ed Stein B. E. (Cambridge, MA: The MIT Press), 301–317.

[B28] Fujisaki W. Nishida S. (2009). Audio–tactile superiority over visuo–tactile and audio–visual combinations in the temporal resolution of synchrony perception. Exp. Brain Res. 198, 245–259. 10.1007/s00221-009-1870-x 19499212

[B29] Fujisaki W. Nishida S. (2010). A common perceptual temporal limit of binding synchronous inputs across different sensory attributes and modalities. Proc. Biol. Sci. 277, 2281–2290. 10.1098/rspb.2010.0243 20335212 PMC2894908

[B30] Fujisaki W. Shimojo S. Kashino M. Nishida S. (2004). Recalibration of audiovisual simultaneity. Nat. Neurosci. 7, 773–778. 10.1038/nn1268 15195098

[B31] Gebhard J. W. Mowbray G. H. (1959). On discriminating the rate of visual flicker and auditory flutter. Am. J. Psychol. 521–529. 10.2307/1419493 13827044

[B32] Getzmann S. (2007). The effect of brief auditory stimuli on visual apparent motion. Perception 36, 1089–1103. 10.1068/p5741 17844974

[B33] Gleiss S. Kayser C. (2014). Oscillatory mechanisms underlying the enhancement of visual motion perception by multisensory congruency. Neuropsychologia 53, 84–93. 10.1016/j.neuropsychologia.2013.11.005 24262657

[B34] Grefkes C. Fink G. R. (2005). The functional organization of the intraparietal sulcus in humans and monkeys. J. Anat. 207, 3–17. 10.1111/j.1469-7580.2005.00426.x 16011542 PMC1571496

[B35] Hagen M. C. Franzén O. McGlone F. Essick G. Dancer C. Pardo J. V. (2002). Tactile motion activates the human middle temporal/V5 (MT/V5) complex. Eur. J. Neurosci. 16, 957–964. 10.1046/j.1460-9568.2002.02139.x 12372032

[B36] Haijiang Q. Saunders J. A. Stone R. W. Backus B. T. (2006). Demonstration of cue recruitment: change in visual appearance by means of Pavlovian conditioning. Proc. Natl. Acad. Sci. U.S.A. 103, 483–488. 10.1073/pnas.0506728103 16387858 PMC1326158

[B37] Hidaka S. Manaka Y. Teramoto W. Sugita Y. Miyauchi R. Gyoba J.. (2009). Alternation of sound location induces visual motion perception of a static object. PLoS ONE 4:e8188. 10.1371/journal.pone.0008188 19997648 PMC2781159

[B38] Hidaka S. Teramoto W. Keetels M. Vroomen J. (2013). Effect of pitch-space correspondence on sound-induced visual motion perception. Exp. Brain Res. 231, 117–126. 10.1007/s00221-013-3674-2 24030519

[B39] Hidaka S. Teramoto W. Kobayashi M. Sugita Y. (2011a). Sound-contingent visual motion aftereffect. BMC Neurosci. 12:44. 10.1186/1471-2202-12-44 21569617 PMC3118223

[B40] Hidaka S. Teramoto W. Sugita Y. Manaka Y. Sakamoto S. Suzuki Y. (2011b). Auditory motion information drives visual motion perception. PLoS ONE 6:e17499. 10.1371/journal.pone.0017499 21408078 PMC3052321

[B41] Howard I. P. Templeton W. B. (1966). Human Spatial Orientation. New York, NY: Wiley.

[B42] Kafaligonul H. Oluk C. (2015). Audiovisual associations alter the perception of low-level visual motion. Front. Integr. Neurosci. 9:26. 10.3389/fnint.2015.00026 25873869 PMC4379893

[B43] Kawabe T. Miura K. Yamada Y. (2008). Audiovisual tau effect. Acta Psychol. 128, 249–254. 10.1016/j.actpsy.2008.01.004 18328993

[B44] Keetels M. Stekelenburg J. J. (2014). Motor-induced visual motion: hand movements driving visual motion perception. Exp. Brain Res. 232, 2865–2877. 10.1007/s00221-014-3959-0 24820287

[B45] Kim R. Peters M. A. Shams L. (2012). 0+1 > 1: How adding noninformative sound improves performance on a visual task. Psychol. Sci. 23, 6–12. 10.1177/0956797611420662 22127367

[B46] Kim R. S. Seitz A. R. Shams L. (2008). Benefits of stimulus congruency for multisensory facilitation of visual learning. PLoS ONE 3:e1532. 10.1371/journal.pone.0001532 18231612 PMC2211398

[B47] Kitagawa N. Ichihara S. (2002). Hearing visual motion in depth. Nature 416, 172–174. 10.1038/416172a 11894093

[B48] Kobayashi M. Teramoto W. Hidaka S. Sugita Y. (2012a). Indiscriminable sounds determine the direction of visual motion. Sci. Rep. 2:365. 10.1038/srep00365 22511997 PMC3328043

[B49] Kobayashi M. Teramoto W. Hidaka S. Sugita Y. (2012b). Sound frequency and aural selectivity in sound-contingent visual motion aftereffect. PLoS ONE 7:e36803. 10.1371/journal.pone.0036803 22649500 PMC3359318

[B50] Konkle T. Wang Q. Hayward V. Moore C. I. (2009). Motion aftereffects transfer between touch and vision. Curr. Biol. 19, 745–750. 10.1016/j.cub.2009.03.035 19361996 PMC3398123

[B51] Körding K. P. Beierholm U. Ma W. J. Quartz S. Tenenbaum J. B. Shams L. (2007). Causal inference in multisensory perception. PLoS ONE 2:e943. 10.1371/journal.pone.0000943 17895984 PMC1978520

[B52] Krebber M. Harwood J. Spitzer B. Keil J. Senkowski D. (2015). Visuotactile motion congruence enhances gamma-band activity in visual and somatosensory cortices. Neuroimage 117, 160–169. 10.1016/j.neuroimage.2015.05.056 26026813

[B53] Kuang S. Zhang T. (2014). Smelling directions: olfaction modulates ambiguous visual motion perception. Sci. Rep. 4, 5796. 10.1038/srep05796 25052162 PMC4107342

[B54] Leung J. Alais D. Carlile S. (2008). Compression of auditory space during rapid head turns. Proc. Natl. Acad. Sci. U.S.A. 105, 6492–6497. 10.1073/pnas.0710837105 18427118 PMC2359774

[B55] Lewis J. W. Beauchamp M. S. DeYoe E. A. (2000). A comparison of visual and auditory motion processing in human cerebral cortex. Cereb. Cortex. 10, 873–888. 10.1093/cercor/10.9.873 10982748

[B56] Maeda F. Kanai R. Shimojo S. (2004). Changing pitch induced visual motion illusion. Curr. Biol. 14, R990–R991. 10.1016/j.cub.2004.11.018 15589145

[B57] Mateeff S. Hohnsbein J. Noack T. (1985). Dynamic visual capture: apparent auditory motion induced by a moving visual target. Perception 14, 721–727 10.1068/p140721 3837873

[B58] Meyer G. F. Wuerger S. M. (2001). Cross-modal integration of auditory and visual motion signals. Neuroreport 12, 2557–2560. 10.1097/00001756-200108080-00053 11496148

[B59] Michel M. M. Jacobs R. A. (2007). Parameter learning but not structure learning: a Bayesian network model of constraints on early perceptual learning. J. Vis. 7:4. 10.1167/7.1.4 17461672

[B60] Morein-Zamir S. Soto-Faraco S. Kingstone A. (2003). Auditory capture of vision: examining temporal ventriloquism. Cogn. Brain Res. 17, 154–163. 10.1016/S0926-6410(03)00089-2 12763201

[B61] Murray M. M. Wallace M. T. (2011). The neural bases of multisensory processes. Boca Raton, FL: CRC Press. 22593873

[B62] Murray M. Spence C. Harris L. (2013). International multisensory researchfForum 2012 meeting special issue. Multisens. Res. 26, 287–289. 10.1163/22134808-00002416 23964480

[B63] Ogawa A. amd Macaluso E. (2013). Audio–visual interactions for motion perception in depth modulate activity in visual area V3A. Neuroimage 71, 158–167. 10.1016/j.neuroimage.2013.01.012 23333414 PMC3838953

[B64] Parise C. V. Harrar V. Ernst M. O. Spence C. (2013). Cross-correlation between auditory and visual signals promotes multisensory integration. Multisens. Res. 26, 307–316. 10.1163/22134808-00002417 23964482

[B65] Parise C. V. Spence C. Ernst M. O. (2012). When correlation implies causation in multisensory integration. Curr. Biol. 22, 46–49. 10.1016/j.cub.2011.11.039 22177899

[B66] Radeau M. Bertelson P. (1987). Auditory–visual interaction and the timing of inputs. Thomas (1941) revisited. Psychol. Res. 49, 17–22. 10.1007/BF00309198 3615744

[B67] Rizzo M. Nawrot M. Zihl J. (1995). Motion and shape perception in cerebral akinetopsia. Brain 118, 1105–1127. 10.1093/brain/118.5.1105 7496774

[B68] Romei V. Murray M. M. Cappe C. Thut G. (2009). Preperceptual and stimulus-selective enhancement of low-level human visual cortex excitability by sounds. Curr. Biol. 19, 1799–1805. 10.1016/j.cub.2009.09.027 19836243

[B69] Roseboom W. Kawabe T. Nishida S. (2013). Direction of visual apparent motion driven by perceptual organization of cross-modal signals. J. Vis. 13:6. 10.1167/13.1.6 23291646

[B70] Sanabria D. Soto-Faraco S. Spence C. (2005). Spatiotemporal interactions between audition and touch depend on hand posture. Exp. Brain Res. 165, 505–514. 10.1007/s00221-005-2327-5 15942735

[B71] Scheef L. Boecker H. Daamen M. Fehse U. Landsberg M. W. Granath D. O.. (2009). Multimodal motion processing in area V5/MT: evidence from an artificial class of audio-visual events. Brain Res. 1252, 94–104. 10.1016/j.brainres.2008.10.067 19083992

[B72] Schlack A. Albright T. D. (2007). Remembering visual motion: neural correlates of associative plasticity and motion recall in cortical area MT. Neuron 53, 881–890. 10.1016/j.neuron.2007.02.028 17359922

[B73] Seitz A. R. Kim R. Shams L. (2006). Sound facilitates visual learning. Curr. Biol. 16, 1422–1427. 10.1016/j.cub.2006.05.048 16860741

[B74] Seitz A. R. Kim R. van Wassenhove V. Shams L. (2007). Simultaneous and independent acquisition of multisensory and unisensory associations. Perception 36, 1445–1454. 10.1068/p5843 18265827

[B75] Sekuler R. Sekuler A. B. Lau R. (1997). Sound alters visual motion perception. Nature 385, 308. 10.1038/385308a0 9002513

[B76] Shams L. Beierholm U. R. (2010). Causal inference in perception. Trends Cogn. Sci. (Regul. Ed). 14, 425–432. 10.1016/j.tics.2010.07.001 20705502

[B77] Shams L. Kamitani Y. Shimojo S. (2000). Illusions: What you see is what you hear. Nature 408, 788. 10.1038/35048669 11130706

[B78] Soto-Faraco S. Kingstone A. Spence C. (2003). Multisensory contributions to the perception of motion. Neuropsychologia 41, 1847–1862. 10.1016/S0028-3932(03)00185-4 14527547

[B79] Soto-Faraco S. Lyons J. Gazzaniga M. Spence C. Kingstone A. (2002). The ventriloquist in motion: illusory capture of dynamic information across sensory modalities. Brain Res. Cogn. Brain Res. 14, 139–146. 10.1016/S0926-6410(02)00068-X 12063137

[B80] Soto-Faraco S. Spence C. Kingstone A. (2004b). Cross-modal dynamic capture: congruency effects in the perception of motion across sensory modalities. J. Exp. Psychol. Hum. Percept. Perform. 30, 330–345. 10.1037/0096-1523.30.2.330 15053692

[B81] Soto-Faraco S. Spence C. Kingstone A. (2005). Assessing automaticity in the audiovisual integration of motion. Acta Psychol. 118, 71–92. 10.1016/j.actpsy.2004.10.008 15627410

[B82] Soto-Faraco S. Spence C. Lloyd D. Kingstone A. (2004a). Moving multisensory research along motion perception across sensory modalities. Curr. Dir. Psychol. Sci. 13, 29–32. 10.1111/j.0963-7214.2004.01301008.x

[B83] Spence C. (2011). Crossmodal correspondences: a tutorial review. Atten. Percept. Psychophys. 73, 971–995 10.3758/s13414-010-0073-7 21264748

[B84] Spence C. (2013). Just how important is spatial coincidence to multisensory integration? Evaluating the spatial rule. Ann. N. Y. Acad. Sci. 1296, 31–49. 10.1111/nyas.12121 23710729

[B85] Spence C. Driver J. (1997). Audiovisual links in exogenous covert spatial orienting. Percept. Psychophys. 59, 1–22. 10.3758/BF03206843 9038403

[B86] Stein B. E. (2012). The New Handbook of Multisensory Processing. Cambridge, MA: The MIT Press.

[B87] Stein B. E. Meredith M. A. (1993). The Merging of the Senses. Cambridge, MA: The MIT Press.

[B88] Stein B. E. Stanford T. R. (2008). Multisensory integration: current issues from the perspective of the single neuron. Nat. Rev. Neurosci. 9, 255–266. 10.1038/nrn2331 18354398

[B89] Stekelenburg J. J. Vroomen J. (2009). Neural correlates of audiovisual motion capture. Exp. Brain Res. 198, 383–390. 10.1007/s00221-009-1763-z 19296094 PMC2733180

[B90] Sugita Y. Suzuki Y. (2003). Audiovisual perception: implicit estimation of sound-arrival time. Nature 421, 911–911. 10.1038/421911a 12606990

[B91] Tajadura-Jiménez A. Väljamäe A. Toshima I. Kimura T. Tsakiris M. Kitagawa N. (2012). Action sounds recalibrate perceived tactile distance. Curr. Biol. 22, R516–R517. 10.1016/j.cub.2012.04.028 22789996

[B92] Teramoto W. Hidaka S. Sugita Y. (2010a). Sounds move a static visual object. PLoS ONE 5:e12255. 10.1371/journal.pone.0012255 20808861 PMC2924383

[B93] Teramoto W. Kobayashi M. Hidaka S. Sugita Y. (2013). Vision contingent auditory pitch aftereffects. Exp. Brain Res. 229, 97–102. 10.1007/s00221-013-3596-z 23727883

[B94] Teramoto W. Manaka Y. Hidaka S. Sugita Y. Miyauchi R. Sakamoto S.. (2010b). Visual motion perception induced by sounds in vertical plane. Neurosci. Lett. 479, 221–225. 10.1016/j.neulet.2010.05.065 20639000

[B95] Teramoto W. Sakamoto S. Furune F. Gyoba J. Suzuki Y. (2012). Compression of auditory space during forward self-motion. PLoS ONE 7:e39402. 10.1371/journal.pone.0039402 22768076 PMC3387142

[B96] van Dam L. C. Parise C. V. Ernst M. O. (2014). Modeling multisensory integration, in Sensory Integration and the Unity of Consciousness, eds Bennettand D. J. Hill C. S. (Cambridge MA: MIT Press), 209–229.

[B97] Van der Stoep N. Nijboer T. C. W. van der Stigchel S. Spence C. (2015). Multisensory interactions in the depth plane in front and rear space: a review. Neuropsychologia 70, 335–349. 10.1016/j.neuropsychologia.2014.12.007 25498407

[B98] van Kemenade B. M. Seymour K. Wacker E. Spitzer B. Blankenburg F. Sterzer P. (2014). Tactile and visual motion direction processing in hMT+/V5. Neuroimage 84, 420–427. 10.1016/j.neuroimage.2013.09.004 24036354

[B99] Watanabe K. Shimojo S. (2001). When sound affects vision: effects of auditory grouping on visual motion perception. Psych. Sci. 12, 109–116. 10.1111/1467-9280.00319 11340918

[B100] Welch R. B. Warren D. H. (1980). Immediate perceptual response to intersensory discrepancy. Psychol. Bull. 88, 638–667. 10.1037/0033-2909.88.3.638 7003641

[B101] Welch R. B. Warren D. H. (1986). Intersensory interactions, in Handbook of Perception and Human Performance, eds Boff K. R. Kaufman L. Thomas J. P. (New York, NY: Wiley), 25.1–25.36.

[B102] Whitney D. Cavanagh P. (2000). Motion distorts visual space: shifting the perceived position of remote stationary objects. Nat. Neurosci. 3, 954–959. 10.1038/78878 10966628

[B103] Williams D. W. Sekuler R. (1984). Coherent global motion percepts from stochastic local motions. Vis. Res. 24, 55–62. 10.1016/0042-6989(84)90144-5 6695508

[B104] Wuerger S. M. Hofbauer M. Meyer G. F. (2003). The integration of auditory and visual motion signals at threshold. Percept. Psychophys. 65, 1188–1196. 10.3758/BF03194844 14710954

[B105] Yamamoto S. Kitazawa S. (2001). Reversal of subjective temporal order due to arm crossing. Nat. Neurosci. 4, 759–765. 10.1038/89559 11426234

[B106] Zihl J. von Cramon D. Mai N. (1983). Selective disturbance of movement vision after bilateral brain damage. Brain 106, 313–340. 10.1093/brain/106.2.313 6850272

[B107] Zihl J. von Cramon D. Mai N. Schmid C. H. (1991). Disturbance of movement vision after bilateral posterior brain damage. Brain 114, 2235–2252. 10.1093/brain/114.5.2235 1933243

[B108] Zvyagintsev M. Nikolaev A. R. Thönnessen H. Sachs O. Dammers J. Mathiak K. (2009). Spatially congruent visual motion modulates activity of the primary auditory cortex. Exp. Brain Res. 198, 391–402. 10.1007/s00221-009-1830-5 19449155

